# Combination therapy for airflow limitation in COPD

**DOI:** 10.1186/2008-2231-20-6

**Published:** 2012-08-28

**Authors:** Mostafa Ghanei, Leila Hoseini Nezhad, Ali Amini Harandi, Farshid Alaeddini, Majid Shohrati, Jafar Aslani

**Affiliations:** 1 Research Center of Chemical Injuries, Baqiyatallah Medical Sciences University, Tehran, Iran; 2 Shahid Beheshti University of Medical Sciences, Tehran, Iran

**Keywords:** COPD, Macrolide, reversibility, Theophylline, Corticosteroids

## Abstract

**Abstract:**

Background and the purpose of the study

Existing evidence confirms that no pharmacologic agent ameliorates the decline in the lung function or changes the prognosis of chronic obstructive pulmonary disease (COPD). We tried a critical combination therapy for management of COPD.

**Methods:**

Current or past smoker (passive or active) COPD patients with moderate to severe COPD who did not respond to primitive therapy (i.e., oral prednisolone (50 mg in the morning) for 5 days; with Beclomethasone Fort (3 puff q12h, totally 1500 micrograms/day), Salmeterol (2 puffs q12h, 50 micrograms/puff) and ipratropium bromide (4 puffs q8h) for two months, enrolled to study. Furthermore they were received N-Acetylcysteine (1200 mg/daily), Azithromycin (tablet 250 mg/every other day) and Theophylline (100 mg BD).

**Results:**

The study group consisted of 44 men and 4 women, with a mean age and standard deviation of 63.6 ± 12.7 years (range 22–86 years). Thirteen of 48 patients (27.0%) was responder based on 15% increasing in FEV 1 (27.7 ± 7.9) after 6.7 ± 6.1 months (57.9 ± 12.9 year old). There were statistically significant differences in age and smoking between responders and non-responders (P value was 0.05 and 0.04 respectively). There was no difference in emphysema and air trapping between two groups (p = 0.13).

**Conclusion:**

Interestingly considerable proportion of patients with COPD can be reversible using combination drug therapy and patients will greatly benefit from different and synergic action of the drugs. The treatment was more effective in younger patients who smoke less.

## Introduction

Worldwide, Chronic obstructive pulmonary disease (COPD) is the sixth leading cause of death [1] and is the only condition in the top 10 causes of death with an increasing prevalence and mortality [2]. It has been estimated that COPD will become the third leading cause of death worldwide by 2020, and its ranking relative for number of disability-adjusted life-years lost will increase from 12th to 5th [3]. COPD is characterised by slowly progressive development of airflow limitation that is poorly reversible, in sharp contrast to asthma where there is variable airflow obstruction that is usually reversible spontaneously or with treatment. While there have been major advances in the understanding and management of asthma, COPD has been relatively neglected and there are no current therapies that reduce the inevitable progression of this disease.

Currently, oxygen therapy for hypoxemic patients and cigarette-smoking cessation are the only interventions known to alter the natural history of COPD and there are not any drugs to reach this goal till now. Although existing evidence confirms that no pharmacologic agent ameliorates the decline in the lung function or changes the prognosis of COPD, we tried a critical combination drugs for the management for patients suffering with COPD. Based on documented medications in patients with COPD and our recent experience with several patients have raised the main question in our mind about whether combination of essential and conventional therapies have sufficient effect to control COPD and even might reverse underlying pathology in susceptible patients. Furthermore, complementary question was that which patient with what characters would get most benefit from this protocol.

## Materials and methods

In a self controlled clinical trial study, we selected current or past smoker (passive or active) COPD patients who had compatible symptoms (e.g. dyspnea, cough and wheezing) and abnormal pulmonary function tests (PFT’s). Patients were excluded if they had a history of significant occupational or other environmental exposures, connective tissue disease, heart failure (evaluated by echocardiography) irregular rhythms active pneumonia, tuberculosis or thrombotic events. In addition, clinical history of any macrolides in the recent 3 months was considered as exclusion criteria. The protocol of the treatment was explained for the patients and written informed consent (approved by the appropriate Institutional Board Review) was obtained from each patient prior to enrollment. None of the patients was forced to drop out of the study due to the type of treatment protocol or side effects. The study was performed with the approval of the ethics committee board and in accordance with good clinical practice and the Declaration of Helsinki.

They had not respond to the following protocol for two months: oral prednisolone (50 mg in the morning) for 5 days; with Beclomethasone Fort (3 puff q12h, totally 1500 micrograms/day), Salmeterol (2 puffs q12h, 50 micrograms/puff) and ipratropium bromide (4 puffs q8h) for two months, enrolled to study. Furthermore they were received N-Acetylcysteine (effervescent tablet 1200 mg/daily), Azithromycin (tablet 250 mg/every other day) and Theophylline (100 mg BD). All patients were recommended to stop smoking. Undesirable response to therapy was considered as remaining of respiratory sign and symptoms and no prominent changes in PFT. The effectiveness of combination therapy was measured using PFT and physical examination from baseline to time that they respond to therapy (i.e., 15% increasing in FEV_1_).

In patients with gastroesophageal reflux symptoms (i.e., heartburn and regurgitation), Omeprazole (20 mg early in the morning) and Famotidine (40 mg at bed time) and Metoclopramide HCl (5 mg BD) were added to their medications.

### Chest HRCT technique and interpretation

An axial GE Hi-speed advantage CT scanner (GE medical system, FXI-plus) at 120 kVp and 200–250 mAs with 1 mm collimation and 10 mm intervals from proximal trachea to the diaphragm was utilized. The scans were obtained in supine position in deep inspiration and also in deep expiration. The HRCT films were reviewed by three expert radiologists using an eFilm workstation (Merge eFilm).

Air trapping was defined as the presence of a radiolucent region of the lungs on expiratory images. The degree of air trapping was assessed by comparing end-inspiratory and expiratory images at similar anatomic levels and at each of the three levels. Air trapping was evaluated in three of the four anatomic levels in each case: upper lung zone, defined as the level of superior aspect of the aortic arch; middle lung zone, defined as the level of the carina; and lower lung zone, defined as the level 5–10 cm below the carina. By using HRCT, emphysema has been described as presence of low attenuation areas, with ill-defined margins, generally without visible walls, bullae (pseudocysts that contain air, with thin well-defined walls), rarefaction of vessels and distortion of the vasculature and complementary signs of hyperinflation of the lungs [4].

### Pulmonary function testing technique and interpretation

Spirometry was performed according to American Thoracic Society criteria. The forced vital capacity (FVC) and FEV_1_ were measured, under the direction of physicians, using a standard spirometer (MasterScreen IOS; Jaeger/Toennies; Hochberg, Germany). Subjects were seated with a nose clip in place and were asked to perform at least three forced expiratory maneuvers. Both the patients and the technician received visual feedback from a monitor during the test, which was repeated until three technically satisfactory curves with a reproducible contour were obtained.

## Results

Sixty four participants were included at baseline and 48 patients completed the study. The study group consisted of 44 men and 4 women, with a mean age and standard deviation of 63.6 ± 12.7 years (range 22–86 years). Thirteen of 48 (27%) patients had improved symptoms according to PFT’s i.e., 15% increasing in forced expiratory volume in 1 second (FEV_1_) after 6.7 ± 6.1 months. Characteristics of responder and non responder patients are shown in detail in Table 1.

**Table 1:** Characteristics of responder and non responder patients

		**Non-responder**	**Responder**	**P value**
**Number**		35	13	
**Age**		65.7 ± 11.9	57.9 ± 12.9	0.054
**Sex**	*male*	33	11	0.028
	*female*	2	2	
**Time of follow up**		8.8 ± 6.1	6.7 ± 6.1	0.03
**FEV1 changes**		0.94 ± 9.9	27.7 ± 7.9	0.0001
**Smoking**	*Past*	21	3	0.044
	*Current*	13	9	
	*Pack per year*	44.7	25.4	0.03
**HRCT finding**	*Emphysema*	12	2	0.13
	*Air trapping*	2	3	
	*Normal*	4	0	

## Discussion

This study evaluated current approved pharmacotherapy all together in the treatment of moderate to severe COPD and provides insight to a guided approach to the use of different medications in stable COPD. It seems this combination critically could improve signs and symptoms and even it could reverse obstruction in considerable proportion of smokers with COPD. In our setting reversibility of 27% of patients is very interesting. Whereas a new definition of COPD has recently been adopted by the Global Initiative on Obstructive Lung Disease (GOLD): "a disease state characterized by airflow limitation that is not fully reversible [5].

It seems age and smokings are two main factors that have effect on response of patients to therapy. Specifically younger patients have responded significantly more than older ones. As such patients who have smoked either for longer period or more pack per year had lower response to therapy. Also it has been shown HRCT findings had no effect on responsiveness.

Because of several pathways of pathogenicity in COPD, it was supposed that multipurpose therapeutic approach would be effective by modulating almost all known pathways and diminish vicious pathologic circles. However, this strategy would work properly only in specific setting and could not be generalized to all patients. Here we summarized pathogenesis and therapeutic modalities.

Several mechanisms contribute to the pathogenesis of COPD [6]. First, the inhalation of noxious particles such as cigarette smoke causes the influx of inflammatory cells into the airways and lungs, leading to chronic inflammation. The airflow limitation is usually progressive and associated with an abnormal inflammatory response of the lungs to noxious particles and gases [5]. Over the past several years, numerous studies have confirmed the important role of inflammation in the airways and lung parenchyma of COPD. Many have advocated a central pathogenic role of this inflammatory response [7-13].

Second, there is a disruption of the balance between proteolytic and anti-proteolytic molecules in the lungs of COPD patients, resulting in an increased proteolytic activity [14]. This causes the destruction of healthy lung parenchyma, which leads to the development of emphysema. This increase in proteolytic activity may be a consequence of inflammation (release of proteolytic enzymes by inflammatory cells such as macrophages and neutrophils) or may arise from genetic factors (e.g., alpha-1 antitrypsin deficiency).

A third mechanism involved in the pathogenesis of COPD is oxidative stress. There is considerable evidence for increased oxidative stress in COPD [15,16]. It occurs when reactive oxygen species are produced in excess of the antioxidant defense mechanisms [6]. Oxidants are generated in the airways by cigarette smoking or are released from inflammatory leukocytes and epithelial cells. Oxidative stress can lead to four processes (chronic inflammation, proteinase/anti-proteinase imbalance, oxidative stress and disruption of the balance between apoptosis and replenishment of structural cells in the lung might contribute to the destruction of lung tissue in response to cigarette smoke, leading to emphysema) involved in the pathogenesis of COPD. These are not independent mechanisms and several interactions between these processes occur during the development of the disease. The oxidative stress can be suppressed by using anti-oxidant. Whereas epidemiological evidence indicates that reduced dietary intake of antioxidants may be a determinant of COPD and population surveys have linked a low dietary intake of the antioxidant ascorbic acid (vitamin C) with worse lung function [17,18]. Here we used N-Acetylcysteine as an approved antioxidant. Treatment by N-Acetylcysteine increased extracellular total anti-oxidant power and total thiol molecules and also improved intracellular glutathione and the outcome of the patients [19]. Also it can reduce viscosity of secretion in the airway of COPD patients.

### Corticosteroids and theophylline

In patients with COPD, corticosteroids are ineffective in suppressing inflammation, including cytokines, chemokines and proteases [20,21]. In vitro the release of IL-8, TNFa and MMP-9 macrophages from normal subjects and normal smokers are inhibited by corticosteroids, whereas corticosteroids are ineffective in macrophages from patients with COPD [22]. The reasons for resistance to corticosteroids in COPD and to lesser extent macrophages from smokers may be the marked reduction in activity of histone deacetylase (HDAC) [22,23], which is recruited to activated inflammatory genes by glucocorticoid receptors to switch off inflammatory genes [24]. The reduction in HDAC activity in macrophages is correlated with increased secretion of cytokines like TNFa and IL-8 and reduced response to corticosteroids. The reduction of HDAC activity on COPD patients may be mediated through oxidative stress and peroxynitrite formation. Although corticosteroids are not effective in inhibiting the secretion of cytokines and proteases from macrophages, other drugs may have considerable advantages. Theophylline in low concentrations increases HDAC activity in alveolar macrophages and in vitro reverses the steroid resistance induced by oxidative stress [25].

### Macrolides

Increasingly, data are becoming available that macrolides have beneficial effects that are independent of their antibacterial activity. These potent anti-inflammatory effects may be particularly important in chronic inflammatory airway disorders. These latter actions may be of greatest benefit for selected patients with COPD, a disorder characterized by augmented pulmonary inflammation at baseline, frequent bacterial colonization/infection, and recurrent exacerbations of disease which further increase lung inflammation. Among their many activities, macrolides have been shown to exert effects on a wide range of cells including nasal and bronchial epithelial cells, alveolar macrophages, monocytes, eosinophils, neutrophils and lymphocytes. The effect on signaling pathways are including NF-κB and AP-1 [26,27].

Neutrophil elastase has subsequently been shown to have several other actions relevant to its potential role in COPD. It is a potent mucus secretagogue of submucosal gland cells and goblet cells [28,29]. The macrolide antibiotics erythromycin and flurithromycin have also been shown to inhibit NE activity [30] and this might account for their beneficial effect on mucus hypersecretion [31]. Macrolides exert a host of effects that collectively limit tissue damage by neutrophils. In addition to effects on chemoattractants, these include inhibiting their oxidant burst, impairing degranulation, and increasing the rate of neutrophil apoptosis. There is also evidence that macrolides decrease mucus viscosity [32]. Importantly, all these immunomodulatory effects are evident at concentrations attainable clinically by low-dose administration. Hence, macrolides have many properties that could moderate the neutrophilic inflammation central to airway damage, and might also improve aspects of airflow obstruction.

Macrolides also exert diverse actions that suppress microbial virulence factors [33-35]. Given these immunomodulatory properties and unique effects on bacterial pathogens macrolide antimicrobial agents have the potential to serve a unique role in the management of chronic inflammatory lung disorders, including COPD.

### H_2_ receptor antagonist and proton pump inhibitor

In patients with gastroesophageal reflux symptoms the addition of a bedtime H_2_ receptor antagonist to proton pump inhibitor reduces the percentage time of the intragastric pH < 4 and also nocturnal acid breakthrough. H_2_ receptor antagonist should be considered as adjunct therapy in whom greater suppression of gastric acid control is considered desirable [36].

## Conclusion

Our study showed 27% of COPD patients have been improved in FEV_1_ using combination drug therapy and patients will greatly benefit from different and synergic action of the drugs in a 8-month period. It is excellent advantage in treatment of irreversible disease like COPD. The role of chronic combination therapy in these patients requires additional study to better define a series of concerns. Any treatment that can lead to reversibility of airflow limitation even in small proportion has a very critical role to improve the disease. Additional, well-designed, placebo-controlled trials are needed before widespread use of this combination regimen for COPD patients.

## Competing interests

The authors declare that they have no competing interests.

## Authors’ contribution

MG: study designing, patient selection, drug prescription, critical revision of manuscript. LHN: patient recruitment and follow up, scale assessment. AAH: literature review, study designing, proposal writing, supervision, data analysis, manuscript writing. FA: study designing, interim analysis, data analysis. MS: pharmacologic consult, drug provider. JA: supervision, patient selection, drug prescription. All authors read and approved the final manuscript.
